# 10α-Hy­droxy-13-{[4-(4-meth­oxy­phen­yl)piperazin-1-yl]meth­yl}-4,9-dimethyl-3,8,15-trioxatetra­cyclo­[10.3.0.0^2,4^.0^7,9^]penta­decan-14-one

**DOI:** 10.1107/S1600536812005818

**Published:** 2012-02-17

**Authors:** Mohamed Moumou, Ahmed Benharref, Jean Claude Daran, Rachid Outouch, Moha Berraho

**Affiliations:** a Laboratoire de Chimie Bioorganique et Analytique, URAC 22, BP 146, FSTM, Universite’ Hassan II, Mohammedia–Casablanca 20810 Mohammedia, Morocco; b Laboratoire de Chimie Biomoléculaire, Substances Naturelles et Réactivité, URAC 16, Faculté des Sciences Semlalia, BP 2390, Boulevard My Abdellah, 40000 Marrakech, Morocco; c Laboratoire de Chimie de Coordination, 205 Route de Narbonne, 31077 Toulouse Cedex 04, France

## Abstract

The title compound, C_26_H_36_N_2_O_6_, was synthesized from 9α-hy­droxy­parthenolide (9α-hy­droxy-4,8-dimethyl-12-methylen-3,14-dioxa-tricyclo­[9.3.0.0^2,4^]tetra­dec-7-en-13-one), which was isolated from the chloro­form extract of the aerial parts of *Anvillea radiata*. The mol­ecule is built up from fused five- and ten-membered rings with two additional ep­oxy ring systems and a meth­oxy­phenyl­piperazine group as a substituent. The ten-membered ring adopts an approximate chair–chair conformation, while the piperazine ring displays a chair conformation and the five-membered ring shows an envelope conformation with the C atom closest to the hy­droxy group forming the flap. The mol­ecular conformation is determined by an O—H⋯N hydrogen bond between the hy­droxy group and a piperazine N atom. The crystal structure is built up by weak C—H⋯O inter­actions.

## Related literature

For background to the medicinal uses of the plant *Anvillea adiata*, see: Abdel Sattar *et al.* (1996 ▶); El Hassany *et al.* (2004 ▶); Qureshi *et al.* (1990 ▶). For the reactivity of this sesquiterpene, see: Hwang *et al.* (2006 ▶); Neukirch *et al.* (2003 ▶); Neelakantan *et al.* (2009 ▶). For ring puckering parameters, see: Cremer & Pople (1975 ▶). For the synthetic procedure, see: Moumou *et al.* (2010 ▶).
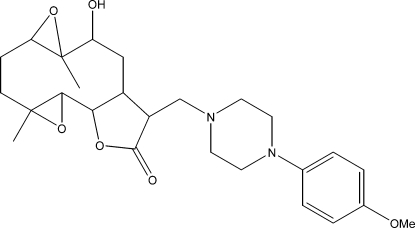


## Experimental

### 

#### Crystal data

C_26_H_36_N_2_O_6_
*M* *_r_* = 472.57
Orthorhombic, 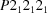

*a* = 8.0770 (7) Å
*b* = 10.2667 (10) Å
*c* = 28.937 (3) Å
*V* = 2399.5 (4) Å^3^
*Z* = 4
Mo *K*α radiation
μ = 0.09 mm^−1^
*T* = 180 K
0.27 × 0.21 × 0.06 mm

#### Data collection

Agilent Xcalibur Sapphire1 long nozzle diffractometer
Absorption correction: multi-scan (*CrysAlis PRO*; Agilent, 2010 ▶) *T*_min_ = 0.732, *T*_max_ = 1.000
14543 measured reflections
2810 independent reflections
1704 reflections with *I* > 2σ(*I*)
*R*_int_ = 0.091

#### Refinement

*R*[*F* ^2^ > 2σ(*F* ^2^)] = 0.072
*wR*(*F* ^2^) = 0.188
*S* = 1.04
2810 reflections
312 parameters
H-atom parameters constrained
Δρ_max_ = 0.29 e Å^−3^
Δρ_min_ = −0.32 e Å^−3^

### 

Data collection: *CrysAlis PRO* (Agilent, 2010 ▶); cell refinement: *CrysAlis PRO*; data reduction: *CrysAlis PRO*; program(s) used to solve structure: *SHELXS97* (Sheldrick, 2008 ▶); program(s) used to refine structure: *SHELXL97* (Sheldrick, 2008 ▶); molecular graphics: *ORTEP-3 for Windows* (Farrugia, 1997 ▶) and *PLATON* (Spek, 2009 ▶); software used to prepare material for publication: *WinGX* (Farrugia, 1999 ▶).

## Supplementary Material

Crystal structure: contains datablock(s) I, global. DOI: 10.1107/S1600536812005818/im2357sup1.cif

Structure factors: contains datablock(s) I. DOI: 10.1107/S1600536812005818/im2357Isup2.hkl

Supplementary material file. DOI: 10.1107/S1600536812005818/im2357Isup3.cml

Additional supplementary materials:  crystallographic information; 3D view; checkCIF report

## Figures and Tables

**Table 1:** Hydrogen-bond geometry (Å, °)

*D*—H⋯*A*	*D*—H	H⋯*A*	*D*⋯*A*	*D*—H⋯*A*
O1—H1⋯N1	0.82	2.10	2.901 (6)	165
C9—H9*B*⋯O1^i^	0.97	2.50	3.345 (7)	145
C14—H14⋯O5^ii^	0.98	2.49	3.447 (7)	165
C15—H15⋯O2^i^	0.98	2.51	3.342 (7)	142
C24—H24⋯O2^i^	0.98	2.33	3.185 (7)	146
C33—H33⋯O3^iii^	0.93	2.53	3.335 (10)	145

Symmetry codes: (i) 
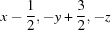
; (ii) 
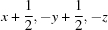
; (iii) 
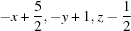
.

**Fig. 1.**
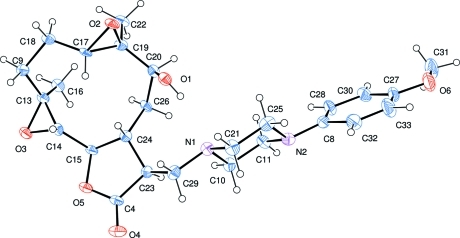
: Molecular structure of the title compound with displacement ellipsoids drawn at the 30% probability level. H atoms are represented as small spheres of arbitrary radii.

**Fig. 2.**
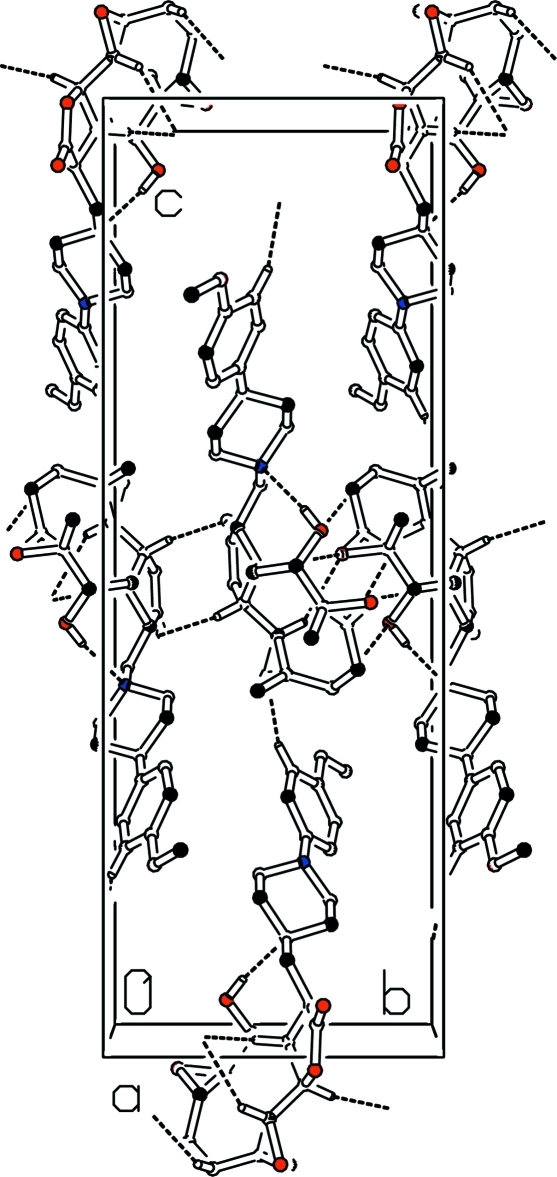
: Partial packing view showing the C–H···O hydrogen bonds as dashed lines. H atoms not involved in hydrogen bonding have been omitted for clarity.

## supplementary crystallographic information

### Comment

The natural sesquiterpene lactone, 9α -hydroxypartenolide is the main constituent of the chloroform extract of the aerial parts of *Anvillea radiata* (El Hassany *et al.*, 2004) and of *Anvillea garcini* (Abdel Sattar *et al.* (1996). The reactivity of this sesquiterpene lactone and its derivatives have been the subject of several studies (Neukirch *et al.*, 2003; Hwang *et al.*, 2006; Neelakantan *et al.*, 2009), in order to prepare products with high value which can be used in the pharmacological industry. In this context, we have synthesed, from 9α-hydroxyparthenolide, the 1β,10α-epoxy-9α-hydoxypartenolide (10α-hydroxy-4,9-dimethyl-13-methylen-3,8,15-dioxa-tetracyclo [10.3.0.0^2,4^.0^7,9^] pentadecan-14-one) (Moumou *et al.*, 2010). This epoxy-hydroxypartenolide treated with one equivalent of 1-(4-methoxyphenyl)-piperazine gives the title compound (I). The crystal structure of (I) is reported herein. The molecule contains a fused ring system and the methoxyphenyl-piperazine group as a substituent to the lactone ring. The molecular structure of (I), Fig.1, shows the lactone ring to adopt an envelope conformation, as indicated by the puckering parameters Q = 0.297 (3) Å and φ =101.7 (8)° (Cremer & Pople, 1975). The ten-membered ring displays an approximate chair-chair conformation, while the piperazine ring has a perfect chair conformation with QT = 0.579 (3) Å, θ = 2.0 (4)° and φ2 =359 (10)°. In the crystal, C—H···O hydrogen bonding links the molecules into sheets lying parallel to the *bc* plane (Table 1, Fig.2). In addition, an intramolecular O1—H1···N1 hydrogen bond is also observed.

### Experimental

The mixture of 1β,10α-epoxy-9α-hydoxypartenolide (10α-hydroxy- 4,9-dimethyl-13-methylen-3,8,15-dioxa-tetracyclo [10.3.0.0^2,4^.0^7,9^] pentadecan-14-one) (500 mg, 1.78 mmol) and one equivalent of 1-(4-methoxyphenyl-piperazine) in EtOH (20 ml) was stirred for twelve hours at room temperature. Then the reaction was stopped by adding water (10 ml) and the solution was extracted with chloroform (3 *x* 20 ml). The combined organic layers were dried over anhydrous MgSO_4_, filtered and concentrated under vacuum to give 730 mg (1.8 mmol) of the title compound (yield: 90%). Recrystallization was performed from in ethyl acetate.

### Refinement

All H atoms were fixed geometrically and treated as riding with C—H = 0.96 Å (methyl), 0.97 Å (methylene), 0.98 Å (methine) with *U*_iso_(H) = 1.2*U*_eq_(methylene, methine) or *U*_iso_(H) = 1.5*U*_eq_(methyl, OH). In the absence of significant anomalous scattering, the absolute configuration could not be reliably determined and thus Friedel pairs were merged and any references to the Flack parameter were removed.

### Crystal data

**Table:**

C_26_H_36_N_2_O_6_	*F*(000) = 1016
*M**_r_* = 472.57	*D*_x_ = 1.308 Mg m^−^^3^
Orthorhombic, *P*2_1_2_1_2_1_	Mo *K*α radiation, λ = 0.71073 Å
Hall symbol: P 2ac 2ab	Cell parameters from 4896 reflections
*a* = 8.0770 (7) Å	θ = 2.9–26.4°
*b* = 10.2667 (10) Å	µ = 0.09 mm^−^^1^
*c* = 28.937 (3) Å	*T* = 180 K
*V* = 2399.5 (4) Å^3^	Platelet, colourless
*Z* = 4	0.27 × 0.21 × 0.06 mm

### Data collection

**Table:**

Agilent Xcalibur Sapphire1 long nozzle diffractometer	2810 independent reflections
Radiation source: fine-focus sealed tube	1704 reflections with *I* > 2σ(*I*)
Graphite monochromator	*R*_int_ = 0.091
Detector resolution: 8.2632 pixels mm^-1^	θ_max_ = 26.4°, θ_min_ = 2.9°
ω scan	*h* = −10→9
Absorption correction: multi-scan (*CrysAlis PRO*; Agilent, 2010)	*k* = −12→12
*T*_min_ = 0.732, *T*_max_ = 1.000	*l* = −35→36
14543 measured reflections	

### Refinement

**Table:**

Refinement on *F*^2^	Secondary atom site location: difference Fourier map
Least-squares matrix: full	Hydrogen site location: inferred from neighbouring sites
*R*[*F*^2^ > 2σ(*F*^2^)] = 0.072	H-atom parameters constrained
*wR*(*F*^2^) = 0.188	*w* = 1/[σ^2^(*F*_o_^2^) + (0.099*P*)^2^] where *P* = (*F*_o_^2^ + 2*F*_c_^2^)/3
*S* = 1.04	(Δ/σ)_max_ = 0.003
2810 reflections	Δρ_max_ = 0.29 e Å^−^^3^
312 parameters	Δρ_min_ = −0.32 e Å^−^^3^
0 restraints	Extinction correction: *SHELXL97* (Sheldrick, 2008), Fc^*^=kFc[1+0.001xFc^2^λ^3^/sin(2θ)]^-1/4^
Primary atom site location: structure-invariant direct methods	Extinction coefficient: 0.015 (3)

### Special details

**Table:**

Experimental. Empirical absorption correction using spherical harmonics, implemented in SCALE3 ABSPACK scaling algorithm. CrysAlisPro (Agilent Technologies)
Geometry. All s.u.'s (except the s.u. in the dihedral angle between two l.s. planes) are estimated using the full covariance matrix. The cell s.u.'s are taken into account individually in the estimation of s.u.'s in distances, angles and torsion angles; correlations between s.u.'s in cell parameters are only used when they are defined by crystal symmetry. An approximate (isotropic) treatment of cell s.u.'s is used for estimating s.u.'s involving l.s. planes.
Refinement. Refinement of *F*^2^ against ALL reflections. The weighted *R*-factor *wR* and goodness of fit *S* are based on *F*^2^, conventional *R*-factors *R* are based on *F*, with *F* set to zero for negative *F*^2^. The threshold expression of *F*^2^ > 2σ(*F*^2^) is used only for calculating *R*-factors(gt) *etc*. and is not relevant to the choice of reflections for refinement. *R*-factors based on *F*^2^ are statistically about twice as large as those based on *F*, and *R*- factors based on ALL data will be even larger.

### Fractional atomic coordinates and isotropic or equivalent isotropic displacement parameters (Å^2^)

**Table:**

	*x*	*y*	*z*	*U*_iso_*/*U*_eq_	
C31	2.1349 (10)	0.2714 (15)	−0.2812 (3)	0.113 (5)	
H31A	2.1795	0.3123	−0.2541	0.169*	
H31B	2.2203	0.2632	−0.3041	0.169*	
H31C	2.0937	0.1866	−0.2734	0.169*	
O4	0.8235 (5)	0.3794 (4)	0.00693 (14)	0.0333 (11)	
O1	1.3161 (5)	0.6411 (4)	−0.04954 (14)	0.0362 (10)	
H1	1.2985	0.5931	−0.0716	0.054*	
O2	1.3704 (5)	0.7880 (4)	0.02594 (14)	0.0356 (10)	
C4	0.8345 (7)	0.3744 (5)	−0.0395 (2)	0.0315 (15)	
O3	0.8874 (5)	0.4797 (4)	0.10678 (14)	0.0365 (11)	
O5	0.7156 (6)	0.3568 (4)	−0.06296 (16)	0.0489 (13)	
N1	1.1958 (6)	0.4608 (5)	−0.11902 (17)	0.0347 (12)	
C8	1.5793 (8)	0.3993 (7)	−0.2180 (2)	0.0376 (15)	
C9	1.0557 (7)	0.6789 (6)	0.1189 (2)	0.0333 (14)	
H9A	1.0636	0.6833	0.1523	0.040*	
H9B	0.9534	0.7212	0.1098	0.040*	
C10	1.2454 (8)	0.3285 (5)	−0.1318 (2)	0.0334 (14)	
H10A	1.1671	0.2935	−0.1540	0.040*	
H10B	1.2433	0.2733	−0.1046	0.040*	
C11	1.4177 (7)	0.3273 (6)	−0.1526 (2)	0.0374 (15)	
H11A	1.4968	0.3577	−0.1298	0.045*	
H11B	1.4472	0.2387	−0.1609	0.045*	
N2	1.4267 (6)	0.4099 (5)	−0.19340 (17)	0.0346 (13)	
C13	1.0497 (7)	0.5390 (6)	0.1044 (2)	0.0296 (14)	
C14	0.9858 (7)	0.4080 (5)	0.0276 (2)	0.0281 (14)	
H14	1.0345	0.3305	0.0419	0.034*	
C15	0.9541 (7)	0.5129 (5)	0.0624 (2)	0.0293 (14)	
H15	0.9042	0.5913	0.0490	0.035*	
C16	1.2353 (7)	0.7204 (5)	0.0486 (2)	0.0287 (14)	
H16	1.1358	0.7098	0.0296	0.034*	
C17	1.1864 (7)	0.4527 (6)	0.1208 (2)	0.0355 (15)	
H17A	1.1843	0.4482	0.1539	0.053*	
H17B	1.2908	0.4875	0.1109	0.053*	
H17C	1.1720	0.3670	0.1082	0.053*	
C18	1.3777 (7)	0.5670 (6)	−0.0123 (2)	0.0322 (14)	
H18	1.4918	0.5425	−0.0199	0.039*	
C19	1.2032 (7)	0.7534 (5)	0.0975 (2)	0.0328 (14)	
H19A	1.1819	0.8462	0.0998	0.039*	
H19B	1.3019	0.7348	0.1154	0.039*	
C20	1.3840 (7)	0.6487 (5)	0.0314 (2)	0.0315 (14)	
C21	1.5243 (8)	0.6110 (7)	0.0626 (2)	0.0424 (17)	
H21A	1.6269	0.6401	0.0494	0.064*	
H21B	1.5266	0.5180	0.0660	0.064*	
H21C	1.5093	0.6508	0.0923	0.064*	
C22	1.2032 (9)	0.5405 (6)	−0.1613 (2)	0.0437 (16)	
H22A	1.1713	0.6292	−0.1540	0.052*	
H22B	1.1249	0.5067	−0.1837	0.052*	
C23	1.0127 (8)	0.3903 (6)	−0.0541 (2)	0.0325 (14)	
H23	1.0620	0.3035	−0.0571	0.039*	
C24	1.0916 (7)	0.4580 (5)	−0.0123 (2)	0.0300 (14)	
H24	1.0704	0.5515	−0.0154	0.036*	
C25	1.3730 (8)	0.5406 (6)	−0.1820 (2)	0.0398 (16)	
H25A	1.3729	0.5936	−0.2098	0.048*	
H25B	1.4504	0.5790	−0.1603	0.048*	
C26	1.2791 (7)	0.4403 (5)	−0.0058 (2)	0.0308 (14)	
H26A	1.3186	0.3760	−0.0277	0.037*	
H26B	1.2996	0.4067	0.0250	0.037*	
C27	1.8662 (8)	0.3621 (8)	−0.2705 (2)	0.051 (2)	
C28	1.7086 (8)	0.3259 (7)	−0.2023 (2)	0.0457 (18)	
H28	1.7012	0.2874	−0.1732	0.055*	
C29	1.0275 (8)	0.4595 (6)	−0.1002 (2)	0.0383 (15)	
H29A	0.9547	0.4172	−0.1223	0.046*	
H29B	0.9899	0.5486	−0.0966	0.046*	
C30	1.8521 (8)	0.3069 (8)	−0.2287 (3)	0.051 (2)	
H30	1.9378	0.2559	−0.2171	0.061*	
O6	2.0017 (7)	0.3498 (8)	−0.29937 (17)	0.086 (2)	
C32	1.5986 (9)	0.4555 (8)	−0.2613 (2)	0.054 (2)	
H32	1.5121	0.5050	−0.2732	0.065*	
C33	1.7365 (10)	0.4416 (10)	−0.2869 (3)	0.072 (3)	
H33	1.7463	0.4840	−0.3152	0.086*	

### Atomic displacement parameters (Å^2^)

**Table:**

	*U*^11^	*U*^22^	*U*^33^	*U*^12^	*U*^13^	*U*^23^
C31	0.031 (5)	0.259 (16)	0.048 (5)	−0.009 (7)	0.004 (4)	−0.012 (8)
O4	0.021 (2)	0.025 (2)	0.053 (3)	−0.0027 (17)	−0.001 (2)	0.0020 (19)
O1	0.032 (2)	0.034 (2)	0.042 (2)	−0.001 (2)	0.003 (2)	0.006 (2)
O2	0.029 (2)	0.025 (2)	0.053 (3)	−0.0114 (18)	0.005 (2)	0.005 (2)
C4	0.020 (3)	0.027 (3)	0.047 (4)	−0.002 (2)	−0.007 (3)	0.003 (3)
O3	0.028 (2)	0.036 (2)	0.045 (3)	−0.0047 (19)	0.005 (2)	0.002 (2)
O5	0.039 (3)	0.040 (3)	0.067 (3)	−0.006 (2)	−0.015 (3)	−0.004 (2)
N1	0.037 (3)	0.028 (3)	0.039 (3)	0.004 (2)	0.001 (3)	0.004 (2)
C8	0.037 (4)	0.046 (4)	0.029 (4)	−0.006 (3)	−0.002 (3)	−0.001 (3)
C9	0.032 (3)	0.026 (3)	0.042 (4)	0.002 (3)	−0.004 (3)	−0.001 (3)
C10	0.042 (4)	0.020 (3)	0.039 (4)	0.010 (3)	0.005 (3)	0.003 (3)
C11	0.033 (4)	0.035 (3)	0.044 (4)	0.000 (3)	0.004 (3)	0.008 (3)
N2	0.037 (3)	0.026 (3)	0.041 (3)	−0.001 (2)	−0.003 (2)	0.005 (2)
C13	0.026 (3)	0.022 (3)	0.041 (4)	0.001 (2)	0.001 (3)	0.002 (3)
C14	0.021 (3)	0.020 (3)	0.044 (4)	−0.001 (2)	−0.001 (3)	0.001 (3)
C15	0.026 (3)	0.023 (3)	0.039 (4)	0.003 (2)	0.008 (3)	0.002 (3)
C16	0.022 (3)	0.021 (3)	0.043 (4)	−0.012 (2)	0.004 (3)	0.005 (3)
C17	0.034 (3)	0.029 (3)	0.044 (4)	0.004 (3)	0.001 (3)	0.010 (3)
C18	0.028 (3)	0.038 (3)	0.030 (3)	0.001 (3)	0.000 (3)	0.006 (3)
C19	0.028 (3)	0.021 (3)	0.049 (4)	−0.002 (2)	−0.002 (3)	0.003 (3)
C20	0.032 (3)	0.020 (3)	0.043 (4)	−0.001 (3)	−0.002 (3)	0.005 (3)
C21	0.025 (3)	0.049 (4)	0.053 (4)	−0.007 (3)	−0.004 (3)	0.002 (3)
C22	0.051 (4)	0.030 (3)	0.050 (4)	0.006 (3)	0.002 (4)	0.005 (3)
C23	0.030 (3)	0.026 (3)	0.041 (4)	0.006 (3)	0.000 (3)	−0.005 (3)
C24	0.032 (3)	0.019 (3)	0.039 (4)	0.001 (3)	0.000 (3)	0.001 (3)
C25	0.055 (4)	0.024 (3)	0.040 (4)	0.005 (3)	−0.008 (3)	0.004 (3)
C26	0.020 (3)	0.026 (3)	0.046 (4)	0.004 (2)	0.005 (3)	0.005 (3)
C27	0.034 (4)	0.085 (6)	0.034 (4)	−0.012 (4)	0.007 (3)	0.001 (4)
C28	0.036 (4)	0.060 (5)	0.041 (4)	−0.003 (4)	0.008 (3)	0.012 (3)
C29	0.041 (4)	0.036 (3)	0.038 (4)	0.010 (3)	−0.002 (3)	−0.001 (3)
C30	0.028 (4)	0.070 (5)	0.056 (5)	0.000 (4)	0.004 (3)	−0.002 (4)
O6	0.047 (4)	0.168 (7)	0.042 (3)	−0.019 (4)	0.014 (3)	0.002 (4)
C32	0.047 (4)	0.077 (5)	0.037 (4)	−0.008 (4)	−0.008 (3)	0.019 (4)
C33	0.054 (5)	0.124 (8)	0.037 (5)	−0.012 (6)	0.005 (4)	0.025 (5)

### Geometric parameters (Å, º)

**Table:**

C31—O6	1.442 (13)	C16—C20	1.494 (8)
C31—H31A	0.9600	C16—H16	0.9800
C31—H31B	0.9600	C17—H17A	0.9600
C31—H31C	0.9600	C17—H17B	0.9600
O4—C4	1.347 (7)	C17—H17C	0.9600
O4—C14	1.470 (7)	C18—C20	1.518 (8)
O1—C18	1.410 (7)	C18—C26	1.537 (8)
O1—H1	0.8200	C18—H18	0.9800
O2—C20	1.443 (7)	C19—H19A	0.9700
O2—C16	1.449 (6)	C19—H19B	0.9700
C4—O5	1.190 (7)	C20—C21	1.500 (9)
C4—C23	1.509 (8)	C21—H21A	0.9600
O3—C15	1.435 (7)	C21—H21B	0.9600
O3—C13	1.447 (7)	C21—H21C	0.9600
N1—C10	1.463 (7)	C22—C25	1.497 (9)
N1—C29	1.464 (8)	C22—H22A	0.9700
N1—C22	1.473 (8)	C22—H22B	0.9700
C8—C28	1.366 (9)	C23—C29	1.517 (8)
C8—C32	1.388 (9)	C23—C24	1.532 (8)
C8—N2	1.428 (8)	C23—H23	0.9800
C9—C13	1.497 (8)	C24—C26	1.537 (8)
C9—C19	1.546 (8)	C24—H24	0.9800
C9—H9A	0.9700	C25—H25A	0.9700
C9—H9B	0.9700	C25—H25B	0.9700
C10—C11	1.516 (8)	C26—H26A	0.9700
C10—H10A	0.9700	C26—H26B	0.9700
C10—H10B	0.9700	C27—C30	1.341 (10)
C11—N2	1.457 (8)	C27—O6	1.383 (8)
C11—H11A	0.9700	C27—C33	1.410 (11)
C11—H11B	0.9700	C28—C30	1.402 (10)
N2—C25	1.448 (8)	C28—H28	0.9300
C13—C15	1.465 (9)	C29—H29A	0.9700
C13—C17	1.493 (8)	C29—H29B	0.9700
C14—C15	1.496 (8)	C30—H30	0.9300
C14—C24	1.526 (8)	C32—C33	1.345 (10)
C14—H14	0.9800	C32—H32	0.9300
C15—H15	0.9800	C33—H33	0.9300
C16—C19	1.477 (8)		
			
O6—C31—H31A	109.5	O1—C18—H18	107.3
O6—C31—H31B	109.5	C20—C18—H18	107.3
H31A—C31—H31B	109.5	C26—C18—H18	107.3
O6—C31—H31C	109.5	C16—C19—C9	113.9 (5)
H31A—C31—H31C	109.5	C16—C19—H19A	108.8
H31B—C31—H31C	109.5	C9—C19—H19A	108.8
C4—O4—C14	110.7 (4)	C16—C19—H19B	108.8
C18—O1—H1	109.5	C9—C19—H19B	108.8
C20—O2—C16	62.2 (4)	H19A—C19—H19B	107.7
O5—C4—O4	121.5 (6)	O2—C20—C16	59.1 (3)
O5—C4—C23	128.8 (6)	O2—C20—C21	112.3 (5)
O4—C4—C23	109.7 (5)	C16—C20—C21	122.3 (5)
C15—O3—C13	61.1 (4)	O2—C20—C18	117.1 (5)
C10—N1—C29	109.9 (5)	C16—C20—C18	121.6 (5)
C10—N1—C22	107.1 (5)	C21—C20—C18	112.5 (5)
C29—N1—C22	110.6 (5)	C20—C21—H21A	109.5
C28—C8—C32	116.4 (6)	C20—C21—H21B	109.5
C28—C8—N2	122.4 (6)	H21A—C21—H21B	109.5
C32—C8—N2	121.0 (6)	C20—C21—H21C	109.5
C13—C9—C19	112.8 (5)	H21A—C21—H21C	109.5
C13—C9—H9A	109.0	H21B—C21—H21C	109.5
C19—C9—H9A	109.0	N1—C22—C25	111.7 (5)
C13—C9—H9B	109.0	N1—C22—H22A	109.3
C19—C9—H9B	109.0	C25—C22—H22A	109.3
H9A—C9—H9B	107.8	N1—C22—H22B	109.3
N1—C10—C11	111.1 (5)	C25—C22—H22B	109.3
N1—C10—H10A	109.4	H22A—C22—H22B	107.9
C11—C10—H10A	109.4	C4—C23—C29	111.8 (5)
N1—C10—H10B	109.4	C4—C23—C24	103.0 (5)
C11—C10—H10B	109.4	C29—C23—C24	116.7 (5)
H10A—C10—H10B	108.0	C4—C23—H23	108.3
N2—C11—C10	111.2 (5)	C29—C23—H23	108.3
N2—C11—H11A	109.4	C24—C23—H23	108.3
C10—C11—H11A	109.4	C14—C24—C23	102.2 (5)
N2—C11—H11B	109.4	C14—C24—C26	114.8 (5)
C10—C11—H11B	109.4	C23—C24—C26	117.0 (5)
H11A—C11—H11B	108.0	C14—C24—H24	107.5
C8—N2—C25	116.3 (5)	C23—C24—H24	107.5
C8—N2—C11	113.8 (5)	C26—C24—H24	107.5
C25—N2—C11	109.9 (5)	N2—C25—C22	111.4 (5)
O3—C13—C15	59.0 (4)	N2—C25—H25A	109.3
O3—C13—C17	113.9 (5)	C22—C25—H25A	109.3
C15—C13—C17	123.0 (5)	N2—C25—H25B	109.3
O3—C13—C9	114.8 (5)	C22—C25—H25B	109.3
C15—C13—C9	115.2 (5)	H25A—C25—H25B	108.0
C17—C13—C9	117.2 (5)	C24—C26—C18	113.3 (5)
O4—C14—C15	105.3 (4)	C24—C26—H26A	108.9
O4—C14—C24	105.0 (4)	C18—C26—H26A	108.9
C15—C14—C24	111.3 (4)	C24—C26—H26B	108.9
O4—C14—H14	111.6	C18—C26—H26B	108.9
C15—C14—H14	111.6	H26A—C26—H26B	107.7
C24—C14—H14	111.6	C30—C27—O6	125.0 (7)
O3—C15—C13	59.8 (4)	C30—C27—C33	119.0 (7)
O3—C15—C14	119.7 (5)	O6—C27—C33	115.9 (7)
C13—C15—C14	126.9 (5)	C8—C28—C30	121.8 (7)
O3—C15—H15	113.3	C8—C28—H28	119.1
C13—C15—H15	113.3	C30—C28—H28	119.1
C14—C15—H15	113.3	N1—C29—C23	113.8 (5)
O2—C16—C19	117.1 (5)	N1—C29—H29A	108.8
O2—C16—C20	58.7 (3)	C23—C29—H29A	108.8
C19—C16—C20	125.0 (5)	N1—C29—H29B	108.8
O2—C16—H16	114.7	C23—C29—H29B	108.8
C19—C16—H16	114.7	H29A—C29—H29B	107.7
C20—C16—H16	114.7	C27—C30—C28	120.2 (7)
C13—C17—H17A	109.5	C27—C30—H30	119.9
C13—C17—H17B	109.5	C28—C30—H30	119.9
H17A—C17—H17B	109.5	C27—O6—C31	114.9 (6)
C13—C17—H17C	109.5	C33—C32—C8	123.1 (7)
H17A—C17—H17C	109.5	C33—C32—H32	118.5
H17B—C17—H17C	109.5	C8—C32—H32	118.5
O1—C18—C20	110.5 (5)	C32—C33—C27	119.4 (7)
O1—C18—C26	111.5 (5)	C32—C33—H33	120.3
C20—C18—C26	112.5 (5)	C27—C33—H33	120.3

### Hydrogen-bond geometry (Å, º)

**Table:**

*D*—H···*A*	*D*—H	H···*A*	*D*···*A*	*D*—H···*A*
O1—H1···N1	0.82	2.10	2.901 (6)	165
C9—H9*B*···O1^i^	0.97	2.50	3.345 (7)	145
C14—H14···O5^ii^	0.98	2.49	3.447 (7)	165
C15—H15···O2^i^	0.98	2.51	3.342 (7)	142
C24—H24···O2^i^	0.98	2.33	3.185 (7)	146
C33—H33···O3^iii^	0.93	2.53	3.335 (10)	145

Symmetry codes: (i) *x*−1/2, −*y*+3/2, −*z*; (ii) *x*+1/2, −*y*+1/2, −*z*; (iii) −*x*+5/2, −*y*+1, *z*−1/2.
